# Reducing unnecessary prescriptions of antibiotics for acute cough: Adaptation of a leaflet aimed at Turkish immigrants in Germany

**DOI:** 10.1186/1471-2296-9-57

**Published:** 2008-10-10

**Authors:** Selime Sahlan, Anja Wollny, Silke Brockmann, Angela Fuchs, Attila Altiner

**Affiliations:** 1 Department of General Practice, University Hospital, Heinrich-Heine-University, 40001 Duesseldorf, Germany

## Abstract

**Background:**

The reduction in the number of unnecessary prescriptions of antibiotics has become one of the most important objectives for primary health care. German GPs report that they are under "pressure to prescribe" antibiotics particularly in consultations with Turkish immigrants. And so a qualitative approach was used to learn more about the socio-medical context of Turkish patients in regard to acute coughs. A German leaflet designed to improve the doctor-patient communication has been positively tested and then adapted for Turkish patients.

**Methods:**

The original leaflet was first translated into Turkish. Then 57 patients belonging to 8 different GPs were interviewed about the leaflet using a semi-standardised script. The material was audio recorded, fully transcribed, and analysed by three independent researchers. As a first step a comprehensive content analysis was performed. Secondly, elements crucial to any Turkish version of the leaflet were identified.

**Results:**

The interviews showed that the leaflets' messages were clearly understood by all patients irrespective of age, gender, and educational background. We identified no major problems in the perception of the translated leaflet but identified several minor points which could be improved. We found that patients were starting to reconsider their attitudes after reading the leaflet.

**Conclusion:**

The leaflet successfully imparted relevant and new information to the target patients. A qualitative approach is a feasible way to prove general acceptance and provides additional information for its adaptation to medico-cultural factors.

## 1. Background

Approximately 80% of all antibiotic prescriptions are issued by GPs. A substantial proportion of these antibiotics are prescribed for acute respiratory infections, and in particular to patients with acute cough.[1-4] A general consensus exists that antibiotics are not usually necessary for the initial treatment of acute cough due to respiratory infections in otherwise healthy adults.[5] The over-prescribing of antibiotics puts patients at risk of side effects, increases the likelihood of bacterial resistance and produces unnecessary costs. As a result the question of when an antibiotic treatment is appropriate has become an important issue for primary health care across Europe, North America and many other countries.[1,6]

Research shows that there are many misconceptions among the general public regarding the effectiveness and appropriate indicators for the use of antibiotics.[7] Furthermore the phenomenon of GPs misinterpreting patient demands has been reported in various primary healthcare centres across Europe.[8-10]

GPs frequently perceive a "pressure to prescribe" antibiotics. As a result they are essentially overestimating the number of patients who are actually demanding antibiotics. Qualitative studies suggest that this overestimation of "patients' prescribing pressure" is largely based on patients' *implicit *concerns about the seriousness of their disease.[11,12] This uneasiness is often misinterpreted by GPs as a demand for antibiotics, and this will eventually result in an antibiotic prescription without any discussion taking place.[8]

These hypotheses were taken into account when the intervention CHANGE (*C*oncerting *H*abits of *A*ntibiotic Prescribing in *Ge*neral Practice) was developed.[13]

We have assumed that if both patients and doctors were informed about the relatively harmless nature of a cough and also the communicative phenomena within the consultation process that lead to the over-prescription of antibiotics, they would be in a position to discuss the issue more openly, improve the quality of the decision-making process, and thus reduce the unnecessary prescribing of antibiotics.

Within the cluster-randomised trial the likelihood of antibiotics being prescribed for acute coughs in otherwise healthy adults was reduced by approximately 40% in the intervention-arm.[14]

The intervention package included a peer-outreach visit performed by a number of specially trained GPs and the provision of specific information material (doctors' brochure, practice poster, patient leaflet) which explicitly addressed the medical and communication issues mentioned above.[13]

After the RCT was completed we conducted open interviews with the participating GPs in order to secure feedback on their experiences of the study.[15] One significant point brought up by the GPs was how valuable the patient leaflets were in other areas of the intervention package. GPs reported that the leaflet helped them to communicate with their patients more effectively, and one GP claimed that it "provided very good information on getting a conversation started". The leaflet did seem to have reduced the perceived pressure to prescribe. One GP reflected "quite often patients just come in and tell me: doctor I just want you to rule out the possibility of something serious".

However, GPs still reported a strong "pressure to prescribe" antibiotics, especially in consultations with Turkish immigrants. For example, one GP told us that ''Turkish immigrants always crave antibiotics [...] because in Turkey antibiotics are in common use".

Based on these GPs' experiences we decided to develop a Turkish version of the leaflet, in the knowledge that Turks are the largest immigrant group in Germany making up 2.5% of the general population, and in many urban regions up to 15%.

One of the most important factors in successful educational interventions in medicine is the adaptation of a leaflet's content to the patients' socio-cultural context and medical beliefs.[16-19] So we decided to employ a qualitative approach in order to develop a version of the leaflet specifically aimed at Turkish immigrants.

## 2. Methods

**Figure 1:**
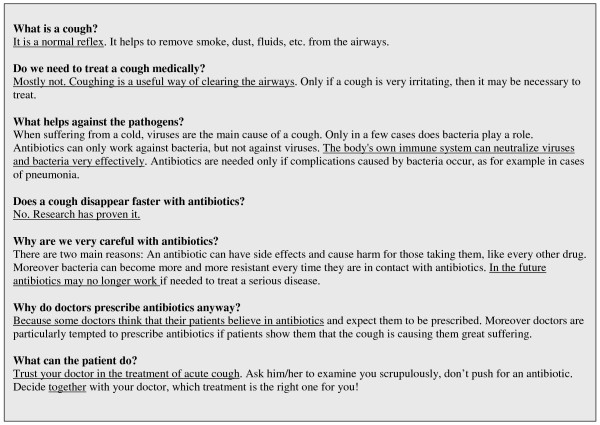
Leaflet before modification.

The leaflet (Fig. 1) was translated into Turkish by a native Turkish speaker (SS) and double-checked using back-translation for linguistic integrity.

We then contacted all 11 Turkish-speaking GPs practicing in three urban areas of North-Rhine Westphalia (Duesseldorf, Dortmund, Essen), an area with 1.9 million inhabitants, of which 9% are Turkish immigrants. 8 GPs consented to having random patients interviewed about the leaflet during regular surgery hours. One researcher (SS) visited each practice for a day and asked Turkish-speaking patients to read the leaflet. Afterwards, the patients were interviewed in Turkish. We ensured that the selection of patients reflected a broad spectrum with regards to age, gender, and socio-economic background. Patients were not selected on the basis that they suffered from an acute cough at the time of the interview.

**Figure 2:**
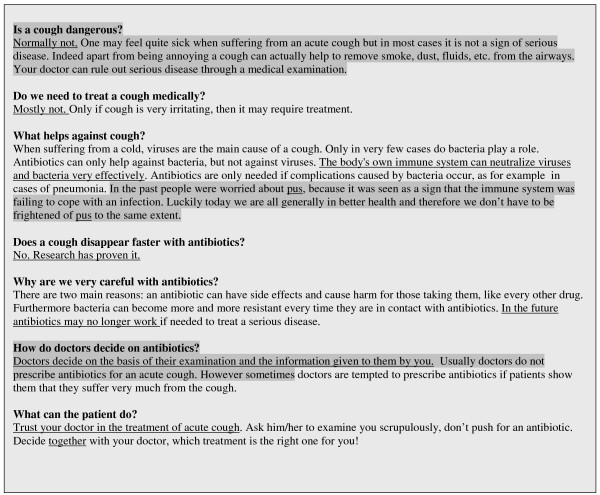
Turkish version of the leaflet after modification, modified phrases marked.

The interviews followed a semi-standardised script using narrative stimuli to encourage open and honest answers. The interviews lasted between 4 and12 minutes (mean average 8 minutes). All interviews were audio-recorded, fully transcribed, and then translated into English. The material was then analysed by three independent researchers combining the perspectives of a Turkish student of medicine (SS), a German GP (AA) and a German health scientist (AW). We analysed the content by categories which focused on how the educational messages within the leaflet were perceived by the patients. Categories were developed inductively until the researchers agreed that satisfactory data saturation had been achieved (after the analysis of 18 selected interviews). All interviews were coded according to these categories. The resulting hypotheses were used to identify areas for consideration in the development of a Turkish version (Fig. 2) of the leaflet.

## 3. Results

### 3.1 Patient Sample

One researcher (SS) visited 8 GP practices. When approaching patients in the waiting rooms the researcher targeted people from a broad range of backgrounds: 57 patients were interviewed (with none refusing to take part); in total, 33 women and 24 men, aged from 17 to 75 years (mean average 39 years) participated. Years of school education ranged from 2 to 13 years (mean average 8 years). The interviewees had between them spent between 1.5 and 39 years in Germany (mean average 26 years), while 6 of the patients had been born in Germany.

### 3.2 Reception of leaflet messages

#### 3.2.1 Cough as a non-threatening symptom

The leaflet emphasised that coughing is a common symptom of an acute upper respiratory infection. Nearly all the Turkish patients spontaneously related to this message. We identified two main types of reaction, either consent or disagreement:

Those patients who were in agreement echoed the leaflet's messages.

Pat 14 (male, 32 years) "I learned that coughing can be helpful and that it should only be treated if one suffers in the extreme."

Pat 40 (female, 23) "Coughing is not a serious illness and coughing throws out microbes."

However, some patients had difficulty with the idea that a cough can be helpful for clearing the throat. Instead they expressed the view that a cough is a harmful disease.

Pat 20 (male, 45) "here it says that coughing may be useful and does not need to be treated [...] but I cannot imagine how a cough can be helpful [...]"

Pat 17 (male, 64) "In here it says that a cough is helpful but it is not. It is harmful. It should be treated as soon as possible..."

#### 3.2.2 Potential harm caused by prescribing antibiotics

A large majority of the interviewed patients acknowledged the potential harm that antibiotics can cause, as mentioned in the leaflet: "I think we use antibiotics to no effect [...] and bacteria have become stronger and stronger" (Pat 28, female, 39).

Some patients were reconsidering their initial views. The following examples show the full range of views – from full agreement to the first signs of a process of reflection.

Pat 16 (male, 17) "I trusted in antibiotics and lots of people do. I thought that antibiotics would cure a cough quicker, but after reading the leaflet I learn that this is not true."

Pat 28 (female, 39) "I didn't know that antibiotics don't treat a cough. I had bronchitis in the past and always took antibiotics [...] I thought that I would recover with the help of antibiotics, but after reading this I am confused. I also learned that the cause of the problem were viruses."

Pat 3 (male, 62) "They [the authors of the leaflet] don't like antibiotics. They think they are not so good. [...] that taking antibiotics would not be beneficial."

Pat 46 (female, 45) "As far as I'm concerned antibiotics do not have any downsides [...] I have never asked until now whether antibiotics have benefits or do harm."

#### 3.2.3 Pus

Although "pus" was not mentioned in the leaflet at all some patients unexpectedly and spontaneously raised this topic. In the context of the interviews, "pus" was somehow understood to be synonymous with the need for antibiotics.

Pat 2 (male, 38) "I've heard that antibiotics work against pus, usually small children are affected by that and then antibiotics make the pus go away."

Pat 1 (male, 32) "As far as I know antibiotics work against pus [...] isn't there pus in our throats when we cough?"

#### 3.2.4. The role of patients and GPs in the decision-making process

A considerable proportion of the interviewees expressed a preference for not taking an active role in the decision-process process.

Pat 5 (male, 45) "I am not a doctor so I don't know how to recover. The doctor would give the recommendation."

Pat 34 (female, 55) "In my opinion patients shouldn't get involved with that. It must be left to the doctors."

Some patients preferred that their involvement be based on "informed consent".

Pat 2 (male, 38) "I would visit my doctor and talk to him [and] I would explain my illness to him. In the end he decides."

On the other hand, some patients, interestingly mostly women, seemed to be more willing to taking an active part in the decision-making process.

Pat 26 (female, 60) "It says in here to decide together with your doctor. That's right. We should decide together with our doctor about treating a cough."

Pat 32 (female, 43) "Patients should clearly state their opinion and doctors should carefully consider it."

Patients also mentioned the relationship between patients' involvement in decision-making and the prescription of antibiotics.

Pat 40 (female, 23) "Of course they [patients] should get involved [...] some doctors are unable to solve the problem any other way so they prescribe antibiotics. They want to escape from the situation."

Patients reacted in very different ways to this supposed misunderstanding with regard to antibiotics. Again, the gender of the interviewee was noteworthy. Men responded with irritation and did not agree with the leaflets' presumption that doctors would prescribe antibiotics because they felt under pressure from their patients rather than for medical reasons.

Pat 5 (male, 49) "That's wrong. It shouldn't be down to patients' demands"

Pat 2 (male, 38) "If doctors know that antibiotics are not useful and unnecessary and still prescribe them, that's not good at all."

Pat 4 (male, 75) "I couldn't imagine that such a thing might happen, however after reading the leaflet I think if it may be possible."

In contrast to this, women displayed a greater understanding and appreciation of these communication issues.

Pat 45 (female, 37) "to push the doctor to prescribe what they want is not good [...] but some doctors prescribe antibiotics just because they think that patients expect it [...] they make this connection based on guesswork."

Pat 43 (female, 29) "the doctor might do it [prescribe an antibiotic] to relax the patient. I can understand that. Because on a psychological level some patients are convinced that they couldn't recover without antibiotics, so they are looking for it."

Pat 44 (female, 58) "I learned not to pressurise the doctor for antibiotics every time, antibiotics are not always useful."

#### 3.2.5. Additional findings

Only a few patients expressed a belief in predestination: "We commit ourselves first to God then to doctors" (Pat 15, male, 42), and otherwise this attitude of "kismet" didn't play an important role in how the patients perceived the leaflet.

When developing the leaflet we found that the discharge of mucus and phlegm were very prominent in perceptions of the disease among German patients. However, for Turkish patients these aspects were of less significance. Instead "pus", as described above, was of much more importance.

Great faith in their doctors' professional skills and their ability to make reasonable medical decisions was common among interviewees, especially in older patients.

The majority of interviewees considered acute coughing a condition which requires medical attention – in common with most German patients. Most patients displayed ambivalent attitudes towards antibiotics. Though sometimes the potential side-effects – and even irrational concerns (for example, fear of addiction) – were mentioned.

## 4. Discussion and conclusion

### 4.1 Discussion

The interviews showed that the leaflets' messages were *understood *by all patients irrespective of age, gender, and educational background. We identified no major obstacles to the understanding of the translated leaflet.

We found in many interviews that patients began to re-examine their attitudes after reading the leaflet. Although the interviewees did not always agree with the message, they understood every detail of the information provided, and patients also *applied *the information to their own personal circumstances. Many tried to *integrate *what was sometimes new and unfamiliar information into their existing concepts of disease. Even when this integration did not succeed, patients still considered the messages carefully – the first step towards any process of behavioural change.

### 4.2 Limitations and Strengths

When discussing the limitations of our study we have to bear in mind that we addressed a very focused research question. In terms of narrative interviewing our interviews were quite brief. Consequently they might not have provided sufficient scope for unanticipated topic areas to arise. However, if we consider the research question, the chosen method (conducting semi-structured interviews with patients waiting to see their doctor) seems to be appropriate. We cannot rule out the possibility that patients may have been to some extent uncomfortable with the interview situation and that some answers were influenced by perceptions of what is socially desirable. However, the analysis did not suggest that this was the case. Patients were included in the study irrespective of whether or not they were suffering from an acute cough at the time of the interview. This may have had an impact on some of the answers given by the patients. However, as all patients would have personally experienced coughing and its treatment at some time in their lives we considered all patients in the waiting room to be eligible for our study.

The strengths of the study lie in the open qualitative approach which was free from pre-selected items and the relatively broad cross-section of patients that made possible a comprehensive analysis of any patterns in the patients' receipt of information.

### 4.3 Conclusions and Practice Implications

The leaflet successfully disseminated relevant and unfamiliar information to the patients it was aimed at. A qualitative approach was shown to be a feasible way to investigate general acceptance and was able to provide additional information.

Based on our analysis, we decided to adapt the leaflet in a number of ways.

We chose not to over-emphasize the fact that "coughing can be useful" but instead focused on the message that coughing is not necessarily a sign of a serious disease. We also dealt with the issue of "pus" and decided to modify the original message on decision-making so that it came across as "less provocative".

For a comparison between the original and the modified leaflet, see Fig. 1 and Fig. 2.

As the original leaflet was only one part of a complex interventional package which proved to be effective in reducing unnecessary antibiotic prescriptions, we cannot claim that the Turkish version on its own will be equally effective. Despite all the enthusiasm surrounding a leaflet that addresses issues of communication rather than merely providing biomedical information, one must be aware of the principal limitations of any written material in this context. We realise that the leaflet may be of value in various aspects of doctor/patient communication, but should not be seen as a substitute for communicative-skills training.

Nevertheless we believe that it may help to improve doctor-patient communication in routine consultations involving antibiotics as it may encourage GPs to discuss the topic of antibiotics more openly with their Turkish patients. The leaflet is available for download at  (German version, modified Turkish version, and additional English version).

## Competing interests

The authors declare that they have no competing interests.

## Authors' contributions

SS carried out the interviews and drafted the manuscript together with AA and AW. AA, AW, and SB conceived he study. AF and SS participated in the study design. All authors read and approved the final manuscript. The authors declare that they have no competing interests.

## Pre-publication history

The pre-publication history for this paper can be accessed here:


